# Establishment of a reference interval for high-sensitivity cardiac troponin I in healthy adults from the Sichuan area

**DOI:** 10.1097/MD.0000000000006252

**Published:** 2017-04-07

**Authors:** Shunjun Li, Yue Zuo, Wenfang Huang

**Affiliations:** Department of Laboratory Medicine, Sichuan Academy of Medical Sciences and Sichuan Provincial People's Hospital, Chengdu, China.

**Keywords:** cardiac troponin I, health checkup, healthy individuals, high-sensitivity, reference interval

## Abstract

High-sensitivity cardiac troponin I (hs-cTnI) has been used in the diagnosis and risk stratification of acute myocardial infarction. However, there is no common consensus on an hs-cTnI reference interval for the Chinese population. The aim of this study was to describe the distribution of hs-TnI and establish the 99th percentile reference interval for hs-cTnI in healthy adults from the Sichuan area.

Serum specimens were collected from 1485 healthy adults (731 men and 754 women ranging in age from 18 to 85 years) in Sichuan Provincial People's Hospital. All specimens were divided into 4 groups according to age distribution: 18 to 35 years, 36 to 50 years, 51 to 65 years, and ≥66 years. Specimens were further divided into younger/middle and older-aged groups based on a cut-off age of 50 years. The serum hs-cTnI concentration was determined using the Abbott ARCHITECT STAT hs-cTnI assay.

The serum hs-cTnI concentration increased with age (*P* < 0.05). The 99th percentiles of hsTnI were 28.0 pg/mL among the whole population, 31.1 pg/mL among men, and 22.7 pg/mL among women. The age-dependent 99th percentiles of hs-cTnI in men and women were as follows: 28.8 versus 12.5 pg/mL for 18 to 35 years, 20.4 versus 9.2 pg/mL for 36 to 50 years, 24.2 versus 13.6 pg/mL for 51 to 65 years, and 27.9 versus 32.2 pg/mL for ≥66 years.

The 99th percentile reference interval for hs-cTnI in healthy adults from the Sichuan area was similar to the manufacturer's recommendation. Men had a higher 99th percentile hs-cTnI value than women in the age range of 18 to 65 years.

## 1 Introduction

Cardiovascular disease is the leading cause of morbidity and mortality worldwide. Cardiac troponins are proteins of the contractile apparatus in the heart muscle and are classified as: troponin I, troponin T, and troponin C based on their function. As cardiac troponins are essential components of the contractile apparatus of myocytes, elevation of circulating cardiac troponins concentration reflects injury of the myocardial cells. Cardiac troponins I and T have been identified as the primary biomarkers for the early diagnosis of acute myocardial infarction (AMI)^[1]^ and for risk stratification of patients suspected of having acute coronary syndrome.^[2]^ Cardiac troponin I is a highly specific indicator of myocyte injury. Therefore, measurement of cardiac troponin I is considered to be highly specific for myocardial injury. International guidelines recommend one concentration measurement of cardiac troponins above the 99th percentile for a healthy reference population with a coefficient of variation of 10% as the cut-off value for the diagnosis of AMI.^[3]^ However, 1st-generation troponins assays were not recommended for the biomarkers of choice in the early diagnosis of AMI due to lack of the recommended detection limit and precision.

Cardiac troponins assays can be classified as: low, medium, high, and ultrasensitive according to their sensitivity.^[4]^ In order to detect the minimal release of troponins, a new generation of high-sensitivity troponins assay has already been introduced into clinical practice.^[5]^ High-sensitivity troponins assays can measure troponins concentrations above the limit of detection in ≥50% of healthy individuals, with a ≤10% coefficient of variation at the 99th percentile.^[6]^ As the sensitivity of troponins assays has improved, these new generation assays can detect the minimal release of cardiac troponins below the 99th percentile, which was often undetectable with the conventional troponins assays. The high-sensitivity cardiac troponin I (hs-cTnI) assay is characterized by a detection limit of concentration in the range of picograms of protein per milliliter.^[7]^ Despite the recommended diagnostic cut-off concentrations for high-sensitivity troponin I (hsTnI), a broad range of cut-off values exist across individual laboratories. A major cause of the broad variation of the 99th percentile cut-off values for hs-cTnI is the selection criteria of the reference population.^[8]^ Males and older persons tend to have higher troponin I values than females and younger persons.^[9–11]^ In addition, the increasingly analytical sensitivity of new troponins assays requires validation in the 99th percentile estimation.

The normative reference interval for hs-cTnI used in China was determined for healthy Western populations. Considering ethnic diversity, the cut-off value for the 99th percentile hs-cTnI concentration for a Western population might not be applicable for the Chinese population, and ignoring this issue could result in misleading clinical decision-making. Therefore, establishment of a reference interval for hs-cTnI in healthy Chinese adults is necessary. In this study, we aimed to describe the distribution of hsTnI and establish the 99th percentile local reference interval for hs-cTnI in healthy adults from the Sichuan area.

## 2 Materials and methods

### 2.1 Study population

Between April 17 and April 26, 2015, a total of 1485 health adults seen for check-ups (mean age: 36 ± 13 years) comprising 731 males and 754 females were randomly selected from the Department of Medical Examination Center of Sichuan Provincial People's Hospital. The occupations of the adults spanned government, enterprises, public institutions, schools, and service industries. Randomization was performed by selecting the odd number of the medical record sequence. The inclusion criteria used to define healthy subjects were as follows: normal body mass index 18.5 to 23;^[12]^ no history of cardiovascular disease; normal liver and renal functions; and without major trauma or cardiac medication history within 3 months. Pregnant women, extreme athletes, those with a chronic disorder requiring regular medication, and those with edema, pleural effusion or ascites, muscle atrophy, cardiac insufficiency, urinary obstruction, or a history of diabetes, hyperlipemia, and hypertension were excluded. Participants were categorized based on age into 4 groups: 18 to 35 years, 36 to 50 years, 51 to 65 years, and ≥66 years. In addition, participants were further divided into younger and older-aged groups according to the cut-off age of 50 years. The study protocol was approved by the Ethics Committee of Sichuan Academy of Medical Sciences and Sichuan Provincial People's Hospital and followed the ethical principles of the Declaration of Helsinki. All participants provided written informed consent.

### 2.2 Specimen collection and routine clinical assessment

Three milliliters of blood were obtained from a forearm vein after at least 8 hours of fasting from all participants. After complete coagulation for 120 minutes, blood specimen was centrifuged at 10,000×*g* for 10 minutes within 2 hours of collection. Serum samples were stored at −80 °C in a freezer until analysis. Serum levels of total bilirubin, direct bilirubin, indirect bilirubin, alanine aminotransferase, aspartate aminotransferase, total protein, albumin, globulin, alkaline phosphatase, gamma-glutamyltransferase, urea nitrogen, creatinine, uric acid, glucose, triglyceride, total cholesterol, high density lipoprotein cholesterol, and low density lipoprotein cholesterol were measured by the automated ARCHITECT c2000 (Abbott Diagnostics, Abbott Park, IL) according to standard clinical laboratory procedures. In addition, demographic characteristics, anthropometric data, heart rate, blood pressure, 12-lead electrocardiogram, chest radiography, and echocardiography were recorded.

Blood pressure was measured in the sitting position. Hypertension was defined as a systolic blood pressure ≥140 mm Hg and/or diastolic blood pressure ≥90 mm Hg, or the use of antihypertensive agents. Diabetes was diagnosed as fasting blood glucose level ≥7 mmol/L, or the use of insulin or oral any hypoglycemic agents. Participants with a body mass index >30 m^2^/kg were grouped as obese.

### 2.3 Determination of hs-cTnI

Hs-cTnI was measured with the Abbott ARCHITECT STAT hs-cTnI assay (Abbott Diagnostics) on Abott i2000 Automatic Immune Analyzers. Precision for the assay was assessed based on the document of the CLSI EP5-A2. Coefficients of variation at the 99th percentiles were tested with participant's samples that had been diluted with assay diluent.

### 2.4 Statistical analysis

All analyses were conducted using SPSS 19.0 statistical software (SPSS Inc., Chicago, IL) and MS Excel 2010. Categorical data are expressed as number or percentage. Normally distributed data are presented as mean ± SD. The Kolmogorov–Smirnov test was applied to verify the normal distribution of variables. The 99th percentile upper reference limit (URL) based on age and gender was calculated in accordance with the CLSI EP28-A3c guideline using a nonparametric analysis.^[13]^ The nonparametric Mann–Whitney *U* test was used to compare differences in hs-cTnI values between different groups. A *P*-value < 0.05 was considered as statistically significant.

## 3 Results

### 3.1 Characteristics of the study population

**Figure 1:**
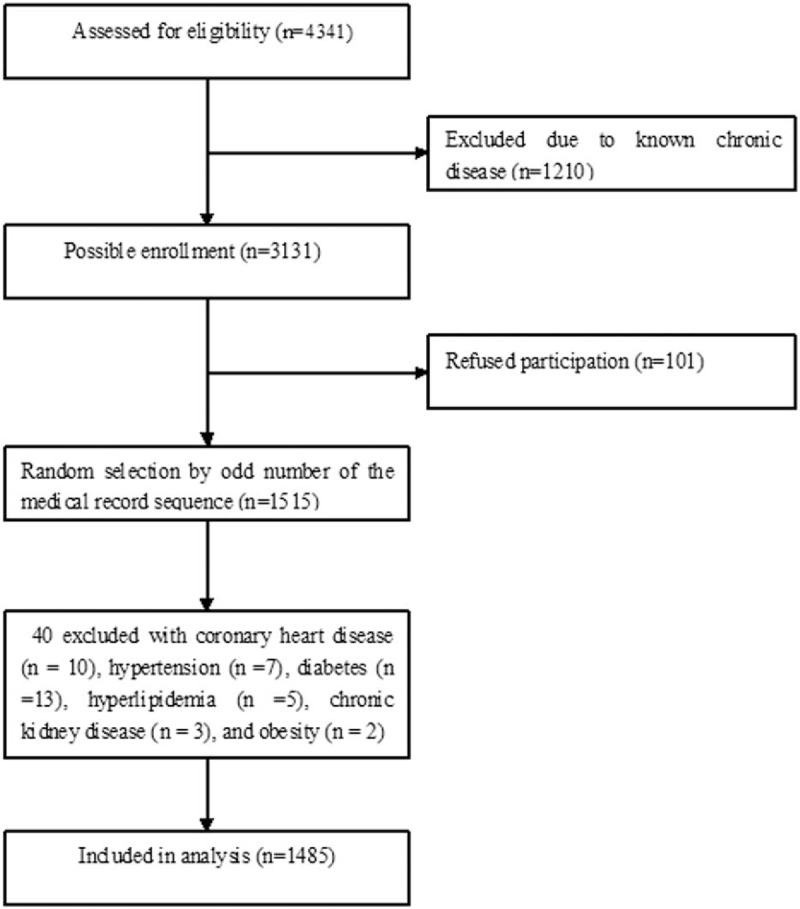
Flow diagram of participants enrollment.

Figure 1 shows the flow diagram of participant enrollment. Of 4341 adults seen for health check-ups, 1210 with known chronic disease were excluded. Of 3131 possible enrollment, 101 adults refused participation. A total of 3030 participants underwent random selection by odd number of the medical record sequence. We further excluded those who presented with coronary heart disease (n = 10), hypertension (n = 7), diabetes (n = 13), hyperlipidemia (n = 5), chronic kidney disease (n = 3), and obesity (n = 2) on the basis of the study inclusion and exclusion criteria. Thus, a total of 1485 healthy adults were enrolled including 731 men (49.2%) and 754 women (50.8%). Participant age varied from 18 to 85 years (mean age, 36 ± 13 years). Among the 731 men, 310 were aged from 15 to 35 years, 226 from 36 to 50 years, 153 from 51 to 65 years, and 42 were ≥66 years. Of the 754 women, 323 were aged from 15 to 35 years, 252 from 36 to 50 years, 144 from 51 to 65 years, and 35 were ≥66 years.

### 3.2 Determination of the 99th percentile for hsTnI

**Figure 2:**
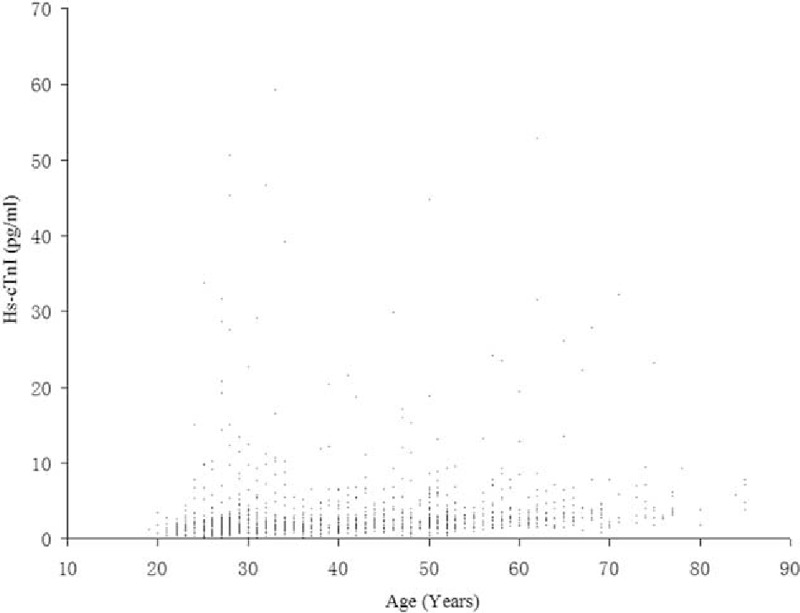
Scatter plot of high-sensitivity troponin I (hsTnI) according to the age distribution.

**Figure 3:**
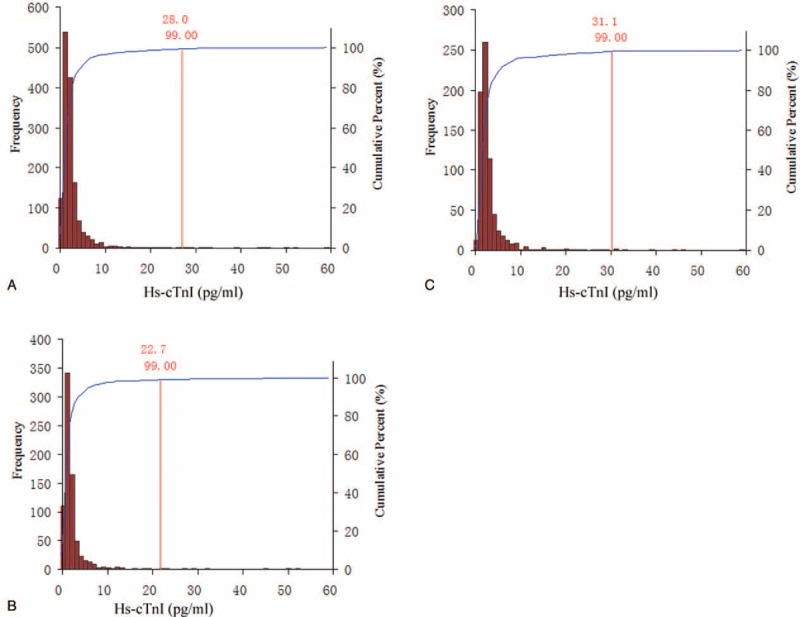
Distribution of high-sensitivity cardiac troponin I (hs-cTnI) concentration in all participants (A), women (B), and men (C). The 99th percentiles were as follows: all participants 28.0 pg/mL (n = 1485), women 22.7 pg/mL (n = 731), and men 31.1 pg/mL (n = 754).

The Kolmogorov–Smirnov test demonstrated the skewed distribution of serum hs-cTnI concentration in different age and gender groups (all *P* *<* 0.05). Figure 2 shows the scatter plot of hsTnI according to the age distribution. The range of measured serum hs-cTnI concentrations in the selected healthy participants was 0.1 to 39.13 pg/mL. As shown in Fig. 3, the 99th percentile URL hs-cTnI concentration was 28.0 pg/mL for all selected healthy adults according to the nonparametric method. The 99th percentile cut-off was significantly higher in men (31.1 pg/mL) than in women (22.7 pg/mL). The male 99 percentile URLs for hs-cTnI concentration were 28.8 pg/mL for ages 18 to 35 years, 20.4 pg/mL for 36 to 50 years, 24.2 pg/mL for 51 to 65 years, and 27.9 pg/mL for age ≥66 years. In women, the 99th percentile URLs for hs-cTnI concentration were 12.5 pg/mL for ages 18 to 35 years, 9.2 pg/mL for 36 to 50 years, 13.6 pg/mL for 51 to 65 years, and 32.2 pg/mL for age ≥66 years. The gender difference in the serum hs-cTnI concentration was more significant for the ages 18 to 65 years. The 99th percentiles for hs-cTnI concentration for the total study population, as well as with the age and gender differences, are summarized in Table 1.

**Table 1:**
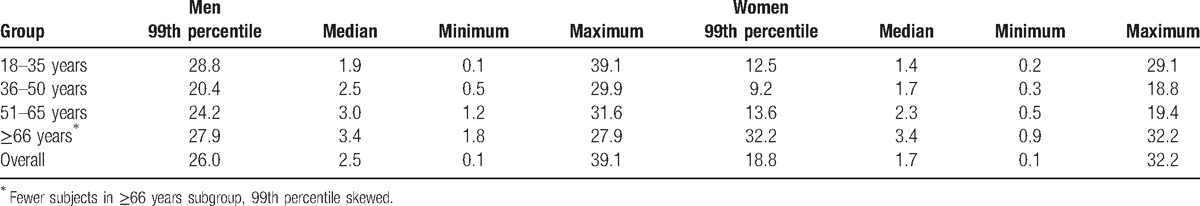
High-sensitivity troponin I concentration (pg/mL) in serum of healthy adults.

### 3.3 Hs-cTnI distribution according to age and gender

**Figure 4:**
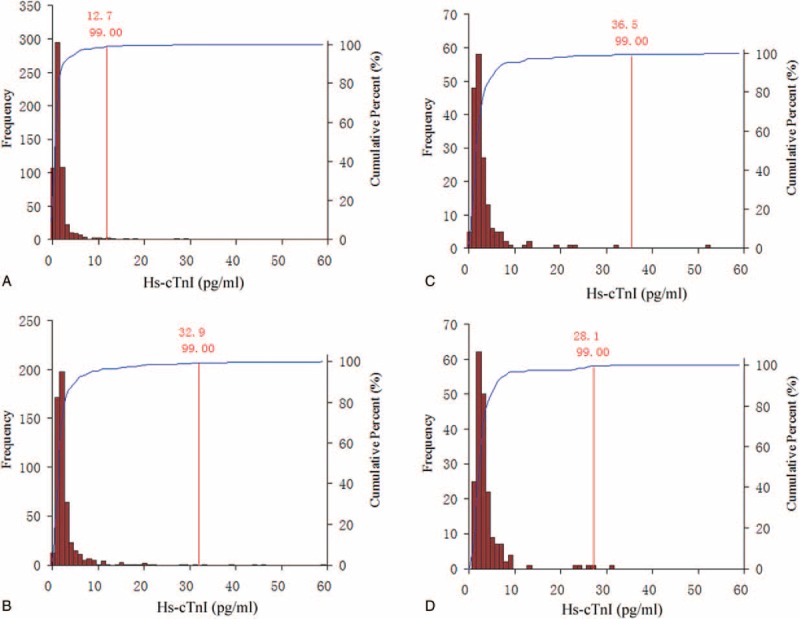
Distribution of high-sensitivity cardiac troponin I (hs-cTnI) concentration in younger and middle-aged women (A), younger and middle-aged men (B), older-aged women (C), and older-aged men (D). The 99th percentiles were as follows: younger and middle-aged women 12.7 pg/mL (n = 574), younger and middle-aged men 32.9 pg/mL (n = 537), older-aged women 36.5 pg/mL (n = 178), and older-aged men group 28.1 pg/mL (n = 194).

The median values of serum hs-cTnI concentration increased with increasing age across the age groups (*P* < 0.05). Men showed a higher serum hs-cTnI concentration than women from age 18 to 65 years (*P* < 0.05). In the younger and middle-aged groups, the serum hs-cTnI concentration was significantly lower in women than in men (*P* < 0.05). By contrast, there was no significant gender difference in the serum hs-cTnI concentration in the older-aged group (*P* > 0.05). The distributions of serum hs-cTnI concentration according to age and gender are shown in Fig. 4.

## 4 Discussion

Estimated biological reference intervals are the most widely used tools in the medical decision-making process. In the current study, we estimated the age- and gender-specific 99th percentile cut-off values using the Abbott hs-cTnI assay in 1487 adults seen for health check-ups from the Sichuan area. We established 28.0 pg/mL as the 99th percentile cut-off value for hs-cTnI in the overall study population. The male and female 99th percentile cut-off values were 31.1 and 22.7 pg/mL, respectively. Specifically, there were significant gender-related differences in the 99th percentile cut-off values for hs-cTnI, with men having a higher concentration from ages 18 to 65 years. However, there were no significant gender differences in the 99th percentile value for hs-cTnI concentration in the 66 years or older subgroup.

Cardiac troponins are blood biomarkers that reflect myocardial cell damage. Assays for cardiac troponin I and T have become widely accepted tools in the early diagnosis of AMI. With the improved sensitivity and precision, the latest troponins assays allow the measurement of hs-cTnI in healthy individuals. Different studies have reported different reference intervals even for the same hs-cTnI assay. Differences in study populations and analytical performance of the assay may largely explain the inconsistent findings of the various studies. For studies on the Abbott hs-cTnI assay in healthy individuals, the 99th percentile cut-off value has ranged from 12.3 to 27 pg/mL.^[14]^ Our overall 99th percentile cut-off value was greater than those reported by Apple et al (23 pg/mL),^[15]^ Zeller et al (27.0 pg/mL),^[11]^ and Aw et al (25.6 pg/mL).^[9]^ The male 99th percentile cut-off value in our study was slightly higher than the value reported by Krintus et al (27 pg/ml),^[16]^ but our female 99th percentile cut-off value was quite different (22.7 vs 11.0 pg/mL). The healthy populations enrolled in these studies were of sufficient sample size to determine reliable overall and gender-specific 99th percentile values. In another Chinese study conducted in Shanghai,^[17]^ the Abbott 99th percentile value for hs-cTnI was 21 pg/mL for all healthy participants, which was lower than the manufacturer's recommendation of 28 pg/mL. This discrepancy may be related to the different age distribution of the participants. Although the 99th percentile cut-off values for hs-cTnI in our study were close to the recommendations of the Western laboratory, establishing the age-, gender-, and ethnic-specific reference intervals for hs-cTnI is necessary.^[18]^

In addition to gender-specific differences, we observed a higher median concentration of hs-cTnI among older individuals. Median values of serum hs-cTnI concentration increased with advancing age. Particularly, we observed a sharply increase in the 99th percentile value for hs-cTnI over 66 years in the female subgroup. This finding was supported by a multicenter study in the Italian population.^[19]^ Eggers et al^[20]^ investigated the hs-cTnI concentrations in 814 community-dwelling individuals at both 70 and 75 years of age, and they found aging resulted in an increase in the 99th percentile for hs-cTnI value, especially in men. These findings suggest that the optimal cut-off value should be higher for adults over 70 years of age than for younger patients when applying hs-cTnI decision thresholds in clinical settings. An increased hs-cTnI concentration should not be interpreted with isolation. As the hs-cTnI concentration was relatively lower in healthy adults, physiological variation of hs-cTnI may affect the disease diagnosis and treatment. The physiological variation and coefficients of variation of hs-cTnI were 18% and 21%, respectively.^[21]^ The clinical implications of implementation of an hs-cTnI assay should be within the context of a patient's medical history and presentation. Apart from at least one concentration of cardiac troponins above the 99th percentile cut-off, an increase or decrease in cardiac troponins concentration is required for the diagnosis of AMI.

The participant selection criteria had a significant impact on the 99th percentile value. However, defining the appropriate healthy population to determine the 99th percentile is challenging. For a study reporting cardiac troponins with the 99th percentile, at least 300 to 500 individuals should be included.^[22]^ Moreover, the reference population should be selected on the basis of a health questionnaire, screening for renal function and ventricular dysfunction. In this study, we selected 1485 young and elderly adults from the population seeking a routine health check-up who met the criteria of international guidelines.

Several limitations of this study should be mentioned. First, individuals with subclinical coronary heart disease could not be excluded based on the electrocardiogram and echocardiography. In addition, natriuretic peptides as a surrogate marker for underlying myocardial dysfunction were not investigated in this study. Second, the relatively small sample size in the aged 66 years or older category could not satisfy the recommended minimum number of 300 participants for subgroup analysis,^[23]^ making it unreliable to examine the effect of age on the hs-cTnI concentration. Finally, this study was purely an analytical evaluation of the hs-cTnI assay. Therefore, diagnostic performance of the Abbott hs-cTnI assay needs to be further investigated.

## 5 Conclusions

Using the Abbott hs-cTnI assay, we established 28.0 pg/mL as the 99th percentile value for hs-cTnI in healthy adults from the Sichuan area; this value was close to the manufacturer's recommendation. Men had a higher 99th percentile hs-cTnI value than women at ages 18 to 65 years. The current study supports the notion that individual laboratories need to establish their own age- and gender-specific threshold values for hs-cTnI when defining reference intervals. Establishing a reference interval for hs-cTnI that is suitable for the healthy Chinese population may improve medical decision-making.
